# Three-dimensional magnetic resonance volumetry of the pituitary gland is effective in detecting short stature in children

**DOI:** 10.3892/etm.2014.1778

**Published:** 2014-06-11

**Authors:** XUE HAN, JIANJUN XIU, ZHAOQIN HUANG, JIE ZHANG, ZHONGHE ZHANG, YIN DONG, XIANSHUN YUAN, QINGWEI LIU

**Affiliations:** Department of Radiology, Shandong Provincial Hospital of Shandong University, Jinan, Shandong 250021, P.R. China

**Keywords:** three-dimensional volumetry, pituitary volume, magnetic resonance imaging, growth hormone deficiency, idiopathic short stature

## Abstract

The aim of the present study was to obtain standard reference values for the pituitary gland volumes of healthy children and to analyze the potential diagnostic values of pituitary gland volumetry for growth hormone deficiency (GHD) and idiopathic short stature (ISS). The volume of the pituitary gland was measured using a thin-section three-dimensional (3D) magnetic resonance imaging (MRI) sequence of magnetization-prepared rapid gradient echo imaging with a section thickness of 1 mm. A group of 75 healthy children aged between 1 and 19 years were recruited to obtain normal volumetry values of the pituitary gland. These individuals demonstrated no evidence of abnormalities to the central nervous or endocrine systems prior to the study. An additional group of 55 children with GHD (n=32) or ISS (n=23) aged between 0 and 14 years were included in the measurement of pituitary gland volume and height. The Student’s t-test was used to evaluate the repetition test, while Pearson’s correlation coefficient and regression analyses were performed to examine the correlations between the volume and height of the pituitary glands. Pituitary gland volume and height demonstrated an increasing trend with age in the healthy children. In addition, the pituitary gland volume exhibited a growth spurt in the early teenage years (10–14 years-old), which was more prominent in females. The growth spurt was not observed for pituitary gland height. When compared with the healthy children, 65.6% of the children with GHD and 34.8% of the children with ISS had smaller pituitary gland volumes. Similarly, 37.5% of the children with GHD and 26.1% of the children with ISS had a smaller pituitary gland height compared with the healthy children. The pituitary gland volume performed significantly better compared with height with regard to the detection rate. Therefore, the results indicated that 3D MRI volumetry was useful for understanding the developmental characteristics of the pituitary gland in healthy children, and that the reference data provided by 3D MRI were effective in the diagnosis of short stature following associations with neuroimaging and clinical functional abnormalities of the pituitary gland.

## Introduction

Growth hormone deficiency (GHD), caused by problems arising in the pituitary gland, is a medical condition in which the body does not produce sufficient growth hormone (GH). GH is a polypeptide hormone that stimulates growth and cell reproduction. Idiopathic short stature (ISS) may be one of the causes of short stature (1). This condition refers to short children without an identifiable disorder of the GH/insulin-like growth factor axis or other endocrine, genetic or organ system disorders (2). GHD is associated with a marked variety of neuroanatomical abnormalities, including a hypoplastic pituitary gland, as identified by magnetic resonance imaging (MRI). Neuroimaging has become an essential part of the diagnostic process for children with GHD in measuring gland size due to the excellent contrast and high spatial resolution (3,4). Currently, the majority of pituitary gland measurements are focused on height, which is considered to be the standard indicator for pituitary gland size (5,6). However, the size and shape of the normal pituitary gland vary considerably and are also affected by age, gender and the hormonal environment (5–10). The variation in shape of the pituitary gland between individuals means that any assessment of size is likely to be subject to a high degree of imprecision unless a true volume is measured (11).

Previously, studies directly measured and indirectly calculated pituitary gland volumes using three-dimensional (3D) volumetry (11–14) and two-dimensional (2D) thin-slice MRI, respectively, for more precise assessments (15,16). Fink *et al* recommended that one-dimensional (height) and indirect (2D) estimations of pituitary gland size and volume should be replaced by direct volumetric analysis (11). However, only a few studies (12) have focused on adolescents or children with short stature.

In the present retrospective study, high-field strength, high-resolution and thin-section 3D MRI sequences were applied to directly measure the volume and height of the pituitary gland in healthy children and children with GHD or ISS. The volumetry values in the assessment of pituitary gland size and in the diagnosis of pituitary gland lesions were investigated.

## Materials and methods

### MRI acquisition and volumetric measurements

MRI was performed using a 3.0-T system with an eight-channel quadrature head coil (MAGNETOM Verio; Siemens, Munich, Germany). Thin-section volumetric studies were conducted with a sequence of magnetization-prepared rapid gradient echo imaging. The following parameters were used: Repetition time/excitation, 1,900 msec/2.45 msec/1; section thickness, 1 mm; inversion time, 900 msec; flip angle, 9°; field of view, 250 mm; and matrix, 256 × 246. The total imaging time was 4 min 18 sec.

**Figure 1:**
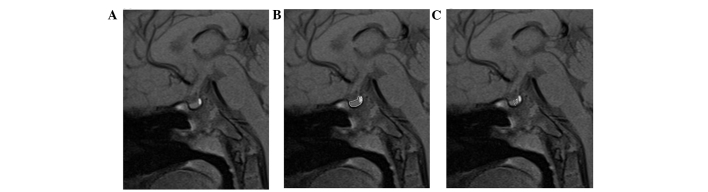
Midline sagittal images of a six year-old female. (A and B) Images derived from the sequence of magnetization-prepared rapid gradient echo imaging. The outline in (B) was determined by manual tracing using a mouse-guided cursor. Regions that did not include the pituitary stalk, but included the neurohypophysis, indicated the pituitary gland, from which the volume of the pituitary gland was calculated using the section thickness and ROIs for every layer. (C) Height measurement of the pituitary gland was calculated as the straight-line distance from the adenohypophysis midpoint of the upper edge to the edge of the gland in sella turcica bottom. The height of the pituitary gland was 0.4 cm in this subject. ROIs, regions of interest.

MRI scans were processed with a Syngo MAGNETOM Verio system (Siemens). In all the cases, the volume was measured on the sagittal image as the boundary is simple to define in this orientation. The regions of interest (ROI) were determined layer-by-layer with manual tracing using a mouse-guided cursor (Fig. 1A and B). The regions did not include the pituitary stalk, but included the neurohypophysis. The volume of the pituitary gland was then calculated using the section thickness and the ROI of every layer. The midsagittal height was obtained from the straight-line distance from the adenohypophysis midpoint of the upper edge to the edge of the gland in the sella turcica bottom (Fig. 1C), according to the traditional method described by Fujisawa (17). A comparison was performed between the short stature children and normal children. Pituitary gland volumes below the minimum value of the corresponding normal range were regarded as dysplastic.

Volumetric measurements of the pituitary gland were performed independently by two neuroradiologists and each neuroradiologist measured the pituitary gland volume twice based on the aforementioned method.

### Subjects

A total of 75 Chinese children aged between 1 and 19 years (mean age, 9.39 years; Table I) were recruited. The individuals had no clinical evidence of pituitary gland lesions (intracranial lesions or endocrinological abnormalities) (18), a history of asphyxia or short-term delivery within 35 weeks. No abnormal observations were identified on routine MRI examination.

A total of 55 Chinese children with short stature were included in the study, with ages ranging between 0 and 14 years (mean age, 8.6 years; Table II). These children were further divided into two groups. Group 1 included 32 children with GHD, while group 2 consisted of 23 children with ISS. The inclusion criteria for children with GHD were as follows: i) Height was below the third percentile among children of the same age and gender; ii) growth rate was <4 cm/year; iii) bone age lagged behind the actual age by two years (Greulich and Pyle standards); iv) serum GH peak was <10 μg/l when stimulated with drugs (clonidine and levodopa) in the GH secretion test; v) levels of serum thyroxine, triiodothyronine and thyroid stimulating hormone were normal; and vi) patients were not affected by genetic metabolic diseases, chromosomal aberrations or any other diseases. The inclusion criteria for children with ISS were the same as the aforementioned standards, with the exception of a serum GH peak of >10 μg/l when stimulated with clonidine and levodopain in the GH secretion test.

Written informed consent was obtained from all the parents or guardians of the children, and all the experimental procedures in the study were approved by the Ethical Committee of Shandong Provincial Hospital of Shandong University (Jinan, China). All the children underwent brain MRI for sellar evaluation between August 2011 and June 2012.

### Statistical analysis

Statistical analysis was performed using SPSS software version 17.0 (SPPS, Inc., Chicago, IL, USA). P<0.05 was considered to indicate a statistically significant difference. The normal range of the pituitary gland volumes was expressed as the mean ± standard deviation. The Student’s t-test was used to evaluate the repetition test, while Pearson’s correlation coefficient and regression analyses were performed to evaluate the correlations between the volume and height of the pituitary glands.

## Results

### Effect of age on pituitary gland height and size

**Figure 2:**
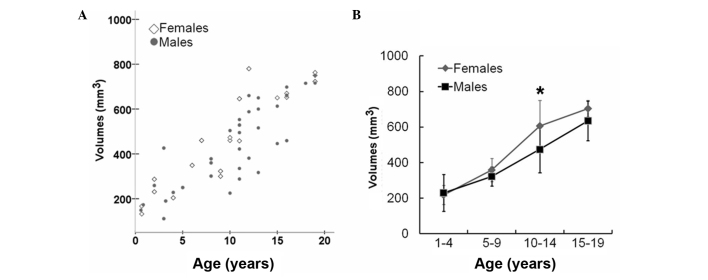
Pituitary gland volumes of children of different ages and genders. (A) Distribution of and (B) average pituitary gland volumes of children of different ages and genders. Data are expressed as the mean ± standard deviation. The pituitary gland exhibited an increasing growth trend in volume with age. A growth spurt in the volume of the pituitary gland was observed in children aged between 10 and 14 years-old, and this trend was more prominent in females. ^*^P<0.05, vs. males.

**Figure 3:**
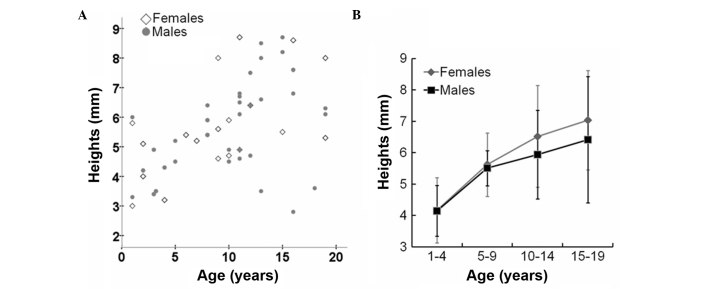
Pituitary gland heights of children of different ages and genders. (A) Distribution of and (B) average pituitary heights of children of different ages and genders. Data are expressed as the mean ± standard deviation. The height of the pituitary gland demonstrated a gradual increase, but without a growth spurt.

To examine the association between pituitary gland volume and height with age, MRI was performed on 75 healthy children. The pituitary gland exhibited an increasing growth trend in volume over age (Fig. 2A and Table III). A growth spurt in the volume of the pituitary gland was observed in children aged between 10 and 14 years-old, and this trend was more prominent in females (P<0.05; Fig. 2B). By contrast, the height of the pituitary gland exhibited a gradual increase without a growth spurt (Fig. 3 and Table III).

The correlation coefficient (r) and adjusted determination coefficient (R^2^) were 0.661 and 0.437, respectively, between the pituitary gland volume and height, as determined by correlation and regression analysis. In the repetition tests, no statistically significant difference was observed between the two measurements of any observer (paired t-test; P=0.164; power of test, 1−β>0.8). Similarly, no statistically significant difference was observed between the measurements of the two observers (P=0.182; power of test, 1−β>0.8).

These observations indicated that the volume of the pituitary gland in normal children increased with age, with a growth spurt between 10 and 14 years of age, whereas the height of the pituitary gland increased gradually without a growth spurt.

### Pituitary gland volume is an improved indicator for GHD and ISS

To investigate the effectiveness of pituitary gland volume and height in detecting GHD or ISS, MRI was conducted on 32 children with GHD and 23 children with ISS. In the 32 children with GHD, 21 individuals had pituitary gland volumes below the minimum value of the corresponding normal range, and the rate of hypoplastic pituitary gland volume was 65.6% (Table IV). In the 23 children with ISS, eight individuals had pituitary gland volumes below the minimum value of the corresponding normal range, and the rate of hypoplastic pituitary gland volume was 34.8% (Table IV). The rate of hypoplastic pituitary gland height was 37.5% for children with GHD and 26.1% for those with ISS (Table IV). These observations demonstrated that the rates of hypoplastic pituitary gland volume and height in children with GHD was higher compared with those in the children with ISS, indicating that pituitary gland volume was a superior indicator for the detection of GHD and ISS.

## Discussion

The size and shape of a normal pituitary gland varies considerably and is affected by age, gender and the hormonal environment (5–10). The pituitary gland size reflects the level of associated hormones in the human body and is important in the diagnosis of pituitary diseases (19). The development of the human body is accompanied by changes to the pituitary gland (20). However, minor changes in pituitary gland height are often difficult to detect as the morphology of the pituitary gland and sella turcica can interfere with accurate measurements. Variations in pituitary gland shape between individuals means that any assessment of pituitary gland size is likely to be subject to a high degree of imprecision unless a true volume is measured (11). Therefore, an increasing number of studies have measured the pituitary gland volume in an attempt to have a more precise assessment of the pituitary gland (12,13,20–22).

Currently, MRI measurements of the pituitary gland volume include 2D geometric methods, voxel-based morphometry and manual surveying and mapping of ROIs. Roldan-Valadez *et al* (21) hypothesized that the traditional geometric method should be replaced by 3D volumetric measurement that had higher accuracy and smaller discrepancy. Voxel-based morphometry is only used to measure the anatomical structure with unclear demarcation, as this technique is poor for the measurement of fine structure, but has the advantages of being simple, saving time and labor. The ROI method may be used for more precise positioning measurements based on anatomical and histological boundaries, and is therefore the *in vivo* measurement closest to the true size of the pituitary gland. Cui *et al* (23) analyzed the pituitary gland volumes in healthy Chinese individuals over the age of 18 years, and their results indicated that 3D MRI clearly demonstrated the morphology and precisely measured the volume of the pituitary gland. However, relatively low MRI field strength was used in their studies, as well as a scanning section thicker than 2 mm and a 2-mm scanning interval. In addition, the authors did not investigate the pituitary gland volume in healthy people aged <18 years-old. Takano *et al* (12) identified that there was a growth spurt in children in the early teenage years, and this spurt was more prominent in females, in a study of 199 healthy Japanese adolescents below the age of 20 years. Similarly, the study by Takano *et al* was also performed with a low MRI field strength. Fink *et al* (11) also demonstrated that assessments using sagittal or coronal data reconstructions produced almost identical results. In the present study, 3D MRI volumetry was used to estimate the pituitary gland volume in healthy children and children with short stature. Firstly, the pituitary gland surface area on each outlined region was determined using the layer-by-layer method on sagittal imaging, from which the volume was calculated by multiplying the surface areas by the thickness of the layers (24).

To the best of our knowledge, there are currently no useful reference data for the normal range of pituitary gland volumes in Chinese children; thus, the present study investigated the pituitary gland volume in healthy children. Only two studies have reported a normal pituitary gland volume in children relative to age. The first study analyzed age-associated pituitary gland volumes in children up to the age of 10, but did not take into account gender differences (11). The other study included prepubertal and postpubertal children, but was limited to the Japanese population (12).

The results obtained from healthy children demonstrated a gradual linear increase in pituitary gland volume over the first ten years of life, which was consistent with the study by Fink *et al* (11). The volume of the pituitary gland exhibited a growth trend with age prior to the age of 20, and there was evidence of a growth spurt in children in the early teenage years (10–14 years old), which was more prominent in females compared with males. These results indicated that the growth of the pituitary gland was more prominent in adolescents, particularly in females. The largest difference in pituitary gland volume was observed between the females and males at the ages of 10–14 years, which was consistent with the studies by Takano *et al* (12). However, the volume of the pituitary gland appeared to differ between Chinese and Japanese adolescents at the ages of 10–14 and 15–19 years. The reasons for the difference may be due to sample sizes or ethnic differences.

The detection rate of hypoplastic pituitary gland volume in children with GHD (65.6%) was higher compared with those with ISS (34.8%). Deficiency of GH secreted by the anterior pituitary gland affects the growth and development of children. MRI volumetry measurements may aid clinicians to diagnose short stature. However, there were also 34.4% of children with GHD demonstrating a normal pituitary gland volume. Therefore, the investigation and evaluation of MRI requires associations with anatomical and functional abnormalities of the pituitary gland. Hypoplastic pituitary gland volume was detected in 34.8% of the children with ISS, indicating that the pituitary gland volume was small in a subgroup of ISS children, although the secretion of GH was normal. However, the mechanisms underlying these observation require further investigation.

In addition, the results of the present study demonstrated that the pituitary gland height exhibited an increasing trend with age in healthy children. The increase in pituitary gland height was moderate in adolescent females, but was slower in males. The growth tendency was different between the pituitary gland height and volume, and the pituitary gland volume performed significantly better than height with regard to the detection rate. In addition, the correlation and regression analyses revealed that the r (0.661) and R^2^ (0.437) values were low between the pituitary gland volume and height. Therefore, the measurement of pituitary gland height should not replace volumetry in the assessment of pituitary gland size due to the imprecision.

In conclusion, 3D MRI volumetry was used in the present study to elucidate the developmental characteristics of the pituitary gland in healthy children. The results indicated that the measurement of pituitary gland height was not able to replace volumetry in the assessment of pituitary gland size. Reference data provided by 3D MRI were valuable in the diagnosis of short stature, however, the evaluation required an association with neuroimaging and clinical functional abnormalities of the pituitary gland. The main limitation of the present study was the small sample size; thus, future, large scale studies are required to determine the clinical utility of these results.

## Figures and Tables

**Table I:** Age and gender distribution of healthy children.

	Age (years)				
		
Participants	1–4	5–9	10–14	15–19	Total
Males (n)	12	8	16	9	45
Females (n)	8	8	7	7	30
Total (n)	20	16	23	16	75

**Table II:** Age and gender distribution of children with GHD or ISS.

	Age (years)						
		
	1–4		5–9		10–14		
				
Condition	Male	Female	Male	Female	Male	Female	Total
GHD (n)	2	4	8	6	9	3	32
ISS (n)	1	2	7	3	10	0	23

GHD, growth hormone deficiency; ISS, idiopathic short stature.

**Table III:** Volumes and heights of the pituitary gland in healthy children (mean ± standard deviation).

	Age (years)							
	
	Male				Female			
		
Parameter	1–4	5–9	10–14	15–19	1–4	5–9	10–14	15–19
Volume (mm^3^)	229.2±104.4	322.3±54.0	474.4±132.0	635.3±111.0	217.9±53.8	358.0±65.6	606.1±144.1	704.4±46.7
Height (mm)	4.15±0.81	5.51±0.56	5.94±1.41	6.41±2.01	4.17±1.04	5.62±1.01	6.52±1.62	7.04±1.58

**Table IV:** Comparison between pituitary volumes and heights in children with GHD or ISS.

	GHD (n)				ISS (n)			
		
Participants	Normal volume	Abnormal volume	Normal height	Abnormal height	Normal volume	Abnormal volume	Normal height	Abnormal height
Age (years)								
1–4	4	2	3	3	2	1	2	1
5–9	5	9	9	5	7	3	8	2
10–14	2	10	8	4	6	4	7	3
Percentage	34.4	65.6	62.5	37.5	65.2	34.8	73.9	26.1

GHD, growth hormone deficiency; ISS, idiopathic short stature. See Table III for the normal sizes pituitary gland volume and height.
